# Supplementary material for: Green Algorithms: Quantifying the Carbon Footprint of Computation
Source: Adv Sci (Weinh). 2021 May 2;8(12):2100707. doi: 10.1002/advs.202100707 (PMC8224424; doi:10.1002/advs.202100707)
Supplement: Supplementary file 1 — Supporting Information [file ADVS-8-2100707-s001.pdf]

© 2021 Wiley-VCH GmbH

ADVANCED SCIENCE
Open Access

Supporting Information

for *Adv. Sci.*, DOI: 10.1002/advs.202100707

Green Algorithms: Quantifying the carbon footprint of computation

*Loïc Lannelongue**, *Jason Grealey,* and *Michael Inouye**

Supporting Information

Green Algorithms: Quantifying the carbon footprint of computation

*Loïc Lannelongue, Jason Grealey, Michael Inouye**

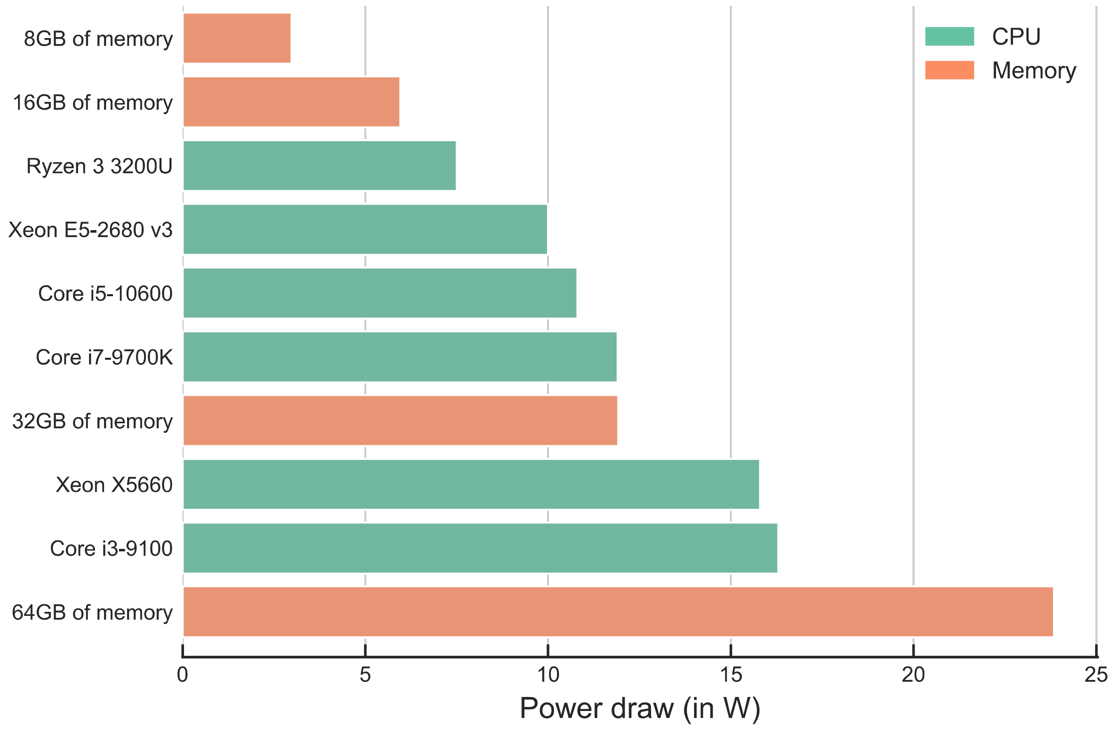

Figure S1: Comparison of power draw (per core) between popular CPUs and memory.

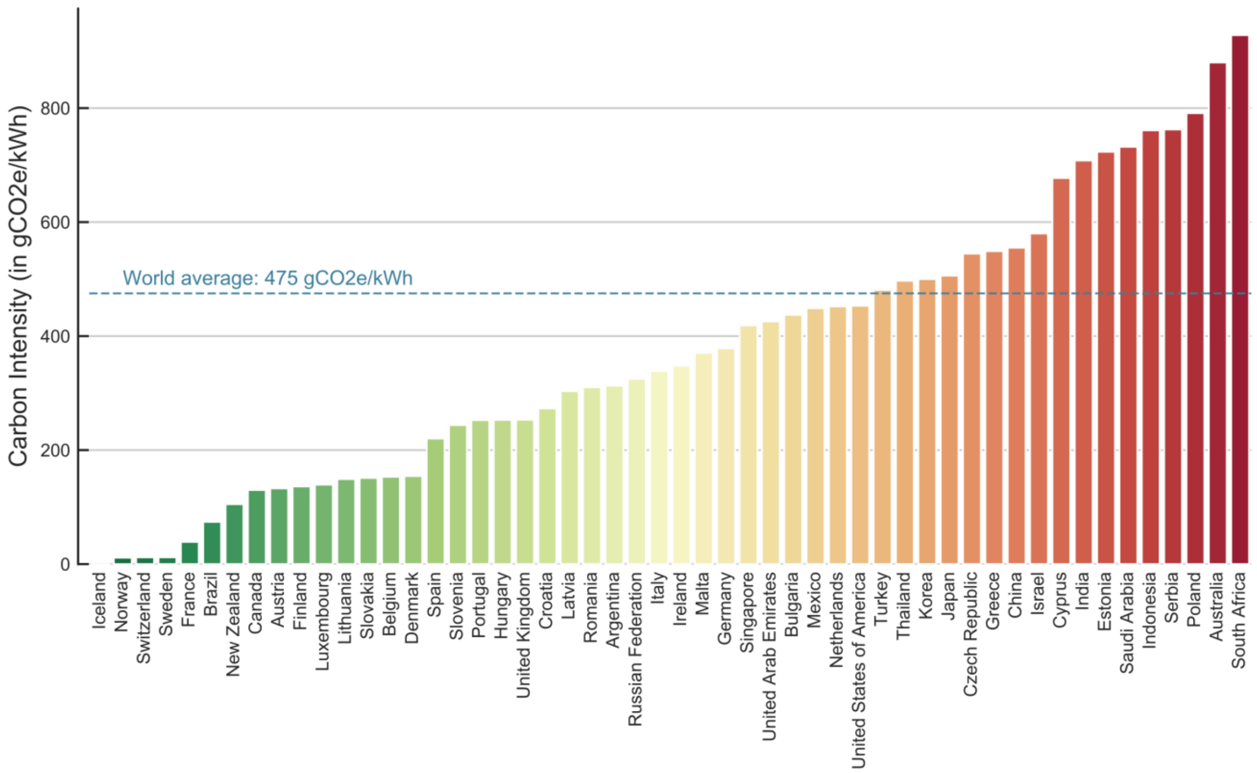

Figure S2: Worldwide carbon intensity distribution by countries, curated from Carbon Footprint[43].

Note S1

We estimated the annual GHG emissions of data centres to be around 100 Mt CO_2e using two distinct approaches. First, using the total electricity demand of data centres, estimated to be around 200 TWh[1] (1% of global demand) and the world average carbon intensity (475 gCO_2e/kWh[49]). Equation (3) (**Methods**) gives a total footprint of 95 x 10^6 tCO_2e. Another estimation is based on data centres being responsible for 0.3% of global emissions[1] (0.3% of 36 x 10^9 tCO_2e[29]), which yields 108 x 10^6 tCO_2e.

Note S2

Shehabi et al.[81] estimate that by 2020, the average capacity of a mechanical hard drive (HDD) in a data centre would be 10TB and the average capacity of a solid-state drive (SSD) 5TB. They also estimate the average power draw to be 6.5W/disk for a HDD and 6W/disk for a SSD. Put together, these numbers give a power draw of 0.65W/TB for a HDD and 1.2W/TB for a SSD. To confirm these numbers, we used experimental benchmarks from Tomes et al.[36] They measure that, in idle mode (i.e. not servicing a request but ready to do so), the power draw of one HDD is 5.75W and 3W for a SSD. In active mode, a HDD draws 8.75W and a SSD 13W; this is coherent with the estimates from Shehabi et al..

These result highlight that the power draw from storage depends on a multitude of parameters, but the order of magnitude can be estimated at 1W per terabyte of data.
